# Does “swapping” maize (*Zea mays* L.) inbred parents affect hybrid grain yield? – a seed production research case study

**DOI:** 10.3389/fpls.2024.1501163

**Published:** 2024-12-20

**Authors:** Patne Nagesh, Satish Ashok Takalkar, Sagala Murali Mohan, Pulime Bhaskara Naidu, Dinesh G. Kanawade, Shyam S. Mandal, Bindiganavile Sampath Vivek

**Affiliations:** ^1^ Global Maize Program, International Maize and Wheat Improvement Center (CIMMYT), Hyderabad, Telangana, India; ^2^ Agricultural Research Station, Dr Panjabrao Deshmukh Vidyapeeth, Buldana, Maharashtra, India; ^3^ Bihar Agriculture College, Bihar Agricultural University, Bhagalpur, Bihar, India

**Keywords:** swapping, synchronization, reciprocal cross, seed production research, seed producibility, nicking

## Abstract

Maize (*Zea mays* L.) is a globally important crop, thriving across diverse environments. Breeding maize inbreds with good combining ability for stable yields under both optimal and stress-prone conditions has been successful. However, to achieve commercial success and impact, seed producibility factors which include female and male parent flowering synchronization and seed parent yield, need to be considered in the early stages of the hybrid development process. In this study, hybrids and their reciprocals were compared through a paired T-test to ascertain if F_1_ performance would be affected by switching (swapping) the roles of the seed and pollen parents. While significant differences were found for grain yield, anthesis days, anthesis silking interval, plant height, ear height, and the number of ears per plant for each group of hybrids and reciprocal crosses, no significant differences were found for hybrids vs. the reciprocals for all of the traits evaluated. This indicated that swapping the roles of female and male parents in successful hybrid combinations does not affect hybrid performance.

## 1 Introduction

Maize (*Zea mays* L.) is an important global crop, thriving in a wide range of soil types and environmental conditions. Modern maize breeding has made it possible to overcome climatic stresses and achieve stable yields in both optimal and stress-prone environments (Rezende et al., 2020). As a first step towards this, understanding the performance and combining ability of inbred lines used as parents in hybrids, is essential (Fan et al., 2014) but it is not the only determinant of a hybrid’s commercial success. Within breeding programs, promising hybrids with good performance are advanced for commercialization but fail to reach their full market sale potential due to a lack of or inadequate understanding of seed production characteristics, namely, synchronization (nicking) between male and female flowering, female parent issues (seed yields, standability, ear rot), male parent issues *vis-à-vis* pollen production (quantum, duration of pollen shed) and dispersal (tassel exertion), ratio of plant height of male parent to ear height of female parent, and optimal female:male row ratios.

In typical maize breeding programs, many inbred lines are crossed with testers in isolation with alternate male rows (normally in a ratio of 3 female:1 male) stagger planted approximately -3 to +5 days apart to capture the maximum variability of inbred lines by ensuring their pollination and seed set for subsequent hybrid evaluation. The seeds of test cross hybrids that are advanced to the next stage of evaluation are then increased either in time-isolations (if logistics and resources permit and if there are a large number of lines) or by hand pollination (if constrained by logistics and resources and if there are fewer lines) by maintaining sufficient staggering between the inbred parents to be crossed. This is why crosses between lines with a large split are made in spite of efforts to reduce advancing hybrids with large splits. Furthermore, inbreds of advanced hybrids are crossed with other elite lines (testers) in the breeding program to evaluate more cross-combinations. This crossing necessarily happens by hand pollination as the numbers of stage-advanced lines to be crossed with multiple testers are much fewer and do not qualify for planting in isolation. In such situations requiring hand pollination, the availability of “sufficient” pollen for obtaining an adequate quantity of seed for a “few” test locations is catered for by planting the parental inbreds with an “approximate stagger”. Due to the relatively large number of hybrids to be advanced, precise studies on the best possible synchronization between the female and male parent (or for that matter, female seed producibility and other seed production traits) cannot be conducted until the hybrid/s have advanced to the commercial consideration (i.e., pre-commercial) stage (generally Stage 5). Until this point, preliminary data on female-male synchronization, female producibility (yield), and other traits are collected on small plots, in breeding locations (and not in seed production locations) and often in breeding nurseries that are to be pollinated. Such data are inadequate for a large seed scale-up.

Maize displays a broad range of flowering times, varying from 35 to 120 days (Colasanti and Muszynski, 2009). In commercial hybrid seed production, achieving synchronization of female and male parents is often challenging, particularly when hybrids have been advanced solely on the basis of F_1_ performance, only to realize at the commercial stage that the parental lines have large maturity differences. Misalignment in flowering periods leads to poor pollination, resulting in reduced seed yield and quality. This is why understanding the genetic and environmental factors that influence the flowering time of each parent is crucial for planning and executing effective seed production. Likewise, all other seed production traits should be considered while assigning the best-suited roles (either female/male) between the two parents of a hybrid. Therefore, before embarking on large-scale commercial hybrid seed production, it is crucial to conduct detailed seed production research (SPR) at commercial seed production sites. While detailed SPR may indicate the best way to use parental lines (or not), quite often maize breeding programs are in a predicament of having to discard a hybrid with poor producibility and nicking that is otherwise compelling in performance and on which resources have been spent in its identification. Hence, as a last resort to retain such a hybrid, it is common for breeders to switch (or swap) the roles of female and male parents i.e., to use the female parent as the male and vice versa, in order to minimize seed production challenges and improve profitability.

Reciprocal effects observed in maize hybrid crosses (F_1_) are known to impact traits such as seed vigor/germination (Mondo et al., 2013; Santos et al., 2017), seedling vigor (Moterle et al., 2011), disease resistance (Zhu et al., 2021), grain yield (John et al., 2024; Fan et al., 2014, 2018), and adaptability to different environmental conditions in hybrid seeds (Jumbo and Carena, 2008; Kovačević et al., 2022). Fleming et al. (1960) reported that hybrids developed using mating designs had significant cytoplasmic effects on days to silking, ear height, plant height, erect plants, and yield. Pollmer et al. (1979) reported that there was no significant variation due to reciprocal differences for protein content and early vigor whereas the variance component due to reciprocal differences was significant only for kernel weight, ear dry matter content, plant height, and ear height. Further, the variance component for the reciprocal × environment interaction was significant for grain yield and days to silking. Dermail et al. (2018) found significant reciprocal cross effects for days to silking, plant height, husked ear length, unhusked yield and husked yield in sweet and waxy corn inbreds crossed in a North Carolina Design II scheme with reciprocals. A failure to consider these reciprocal effects can lead to sub-optimal hybrid performance and the discarding of a hybrid that can otherwise be produced using reciprocal crosses.

The reciprocal studies reported above either used a diallel crossing design or a North Carolina Design II to study the maternal and cytoplasmic effects on grain yield and agronomic traits. Published reports directly comparing hybrids and their reciprocals for grain yield differences using a head-to-head analysis were not found. Given the diverse nature of the studies and their reports, there is an essential need to gain a better understanding of the effects of “swapping” parental lines in tropical maize. The objective of this study was to determine if there were any differences in the performance of grain yield and other traits between hybrids and their respective reciprocals.

## 2 Materials and methods

The seeds of 15 single-cross hybrids identified to have good performance were reconstituted by crossing the parents in the same female and male configurations used in the hybrid identification process (direct crosses). Simultaneously, respective reciprocal hybrids were formed. All crosses were formed in the wet season of 2019. In total, 30 hybrids, along with two commercial checks, were included in this study (**Table 1**). These hybrids were tested across four different locations in India during the dry season of 2019: Buldana in Maharashtra (20°5’N, 76°2’E; 425 m above mean sea level [masl]), Eluru in Andhra Pradesh (16°8’N, 81°1’E; 17.58 masl), and Sabour (25°2’N, 87°0’E; 37.19 masl) and Samastipur (25°9’N, 85°7’E; 48 masl) in Bihar. The trials followed an Alpha (0, 1) lattice design with two replications. Each plot comprised two rows, each 4 m long, with a row spacing of 60 cm and plant-to-plant spacing of 20 cm. The recommended crop management practices for maize were implemented across all test locations to ensure uniform crop stands. Data were collected for field weight (FW), days to anthesis (AD), days to silking (SD), plant height (PH), ear height (EH), number of plants per plot (NPP), number of ears per plot (NEP), and moisture content (MOI) at harvest. Grain yield (GY), anthesis silking interval (ASI), and ears per plant (EPP) were calculated and used in an analysis of variance and for the estimation of various components using META-R software (Multi-Environment Trial Analysis with R) (Alvarado et al., 2015). Two-tailed Student’s T-test between direct and reciprocal crosses (head-to-head analysis) was done to check the hypothesis that there were differences between hybrids formed by direct and reciprocal crosses. Microsoft Excel software in the MS Office 365 package was used for the T-test using best linear unbiased predictors (BLUPs) across the four locations. Homogeneity of variance in both the groups of hybrids was assumed.

**Table 1:** The direct and reciprocal hybrids and two checks evaluated during the dry season in 2019.

Sl. No	Hybrid	Name	Pedigree	Hybrid type
**1**	H1	VH1846A	VL18329×VL111354	Direct cross
**2**	H1	VH1846B	VL111354×VL18329	Reciprocal cross
**3**	H2	VH1899A	VL18329×VL18431	Direct cross
**4**	H2	VH1899B	VL18431×VL18329	Reciprocal cross
**5**	H3	VH1849A	VL18329×VL109378	Direct cross
**6**	H3	VH1849B	VL109378×VL18329	Reciprocal cross
**7**	H4	VH1854A	VL1040×VL18327	Direct cross
**8**	H4	VH1854B	VL18327×VL1040	Reciprocal cross
**9**	H5	VH13729A	VL1110519×VL111354	Direct cross
**10**	H5	VH13729B	VL111354×VL1110519	Reciprocal cross
**11**	H6	VH16224A	VL111354×KL155956	Direct cross
**12**	H6	VH16224B	KL155956×VL111354	Reciprocal cross
**13**	H7	VH1657A	VL154542×VL111354	Direct cross
**14**	H7	VH1657B	VL111354×VL154542	Reciprocal cross
**15**	H8	VH1652A	VL154510×VL111354	Direct cross
**16**	H8	VH1652B	VL111354×VL154510	Reciprocal cross
**17**	H9	VH151703A	VL13853×VL1055	Direct cross
**18**	H9	VH151703B	VL1055×VL13853	Reciprocal cross
**19**	H10	VH122937A	VL119989×VL111354	Direct cross
**20**	H10	VH122937B	VL111354×VL119989	Reciprocal cross
**21**	H11	VH153492A	VL108727×VL111354	Direct cross
**22**	H11	VH153492B	VL111354×VL108727	Reciprocal cross
**23**	H12	VH171219A	VL18448×VL111354	Direct cross
**24**	H12	VH171219B	VL111354×VL18448	Reciprocal cross
**25**	H13	VH18571A	VL18448×VL18329	Direct cross
**26**	H13	VH18571B	VL18329×VL18448	Reciprocal cross
**27**	H14	VH16921A	KL155991×VL1016178	Direct cross
**28**	H14	VH16921B	VL1016178×KL155991	Reciprocal cross
**29**	H15	VH16923A	KL155992×VL1016178	Direct cross
**30**	H15	VH16923B	VL1016178×KL155992	Reciprocal cross
**31**	Check	CAH153	CAH153	CAH153
**32**	Check	NK6240	NK6240	NK6240

## 3 Results and discussion

The analysis of the variance data showed that direct crosses were significant at *p < 0.01* for all traits studied, namely GY, AD, ASI, PH, EH, and EPP across all four locations (**Table 2**). The reciprocal cross differences were significant for GY, AD, PH, and EH at *p < 0.01*. This indicated that there were genotypic differences between entries within each group of direct and reciprocal crosses for the traits studied. The hybrid × location interaction was significant for direct crosses for GY, AD, and ASI while for the reciprocal crosses, GY and EPP were significant. The mean squares for direct vs. reciprocal and direct vs. reciprocal with location were non-significant for all the traits in the study. A paired Student’s T-test at 95% confidence interval showed that the difference between the direct and reciprocal crosses was not significant for any of the traits (**Table 3**). This clearly indicated that switching/swapping between female and male parents of successful hybrid combinations does not affect the performance of the resulting hybrids.

**Table 2:** ANOVA of hybrids evaluated across four locations in India during the dry season in 2019.

Source of variation	DF	Mean squares					
		GY	AD	ASI	PH	EH	EPP
**Rep**	1	18.4**	*0.2ns*	*0.7ns*	1421.1**	232.1*ns*	0.03*ns*
**Loc**	3	126.7**	39504.9**	110.8**	65237.3**	12581.2**	0.04*
**Rep:block**	14	2.1*	2.7ns	0.9*	272.4*ns*	155.6*	0.01
**All crosses**	29	42.5**	16.3**	1.0**	1597.7**	776.0**	0.02*ns*
**Direct crosses**	14	38.7**	28.9**	0.8*	1278.6**	640.6**	0.01*
** Reciprocal crosses**	14	49.1**	4.9**	*1.1ns*	1993.3**	937.1**	0.02*ns*
** Direct vs reciprocal**	1	*3.0ns*	*1.8ns*	*1.6ns*	*528.1ns*	*416.1ns*	0.04*ns*
**All crosses:loc**	87	2.6**	2.8**	0.7*	177.9*ns*	106.6*ns*	0.02**
** Direct crosses:loc**	42	3.4**	3.0*	0.9**	221.7*ns*	114.0*ns*	0.01*ns*
** Reciprocal crosses:loc**	42	2.1**	2.9ns	0.4*ns*	125.5*ns*	106.1*ns*	0.03*
** Direct vs reciprocal:loc**	1	*1.1ns*	*0.2ns*	*3.5ns*	*892.8ns*	31.1*ns*	*0.21ns*
**Residuals**	104	1.1	1.8	0.5	199.7	82.2	0.01

*, ** significant at 0.05 and 0.01 probability levels, respectively; *ns*, nonsignificant; df, degrees of freedom; grain yield, GY; anthesis days, AD; anthesis-silking interval, ASI; plant height, PH; ear height, EH; ears per plant, EPP.

**Table 3:** Mean performance and t-test for grain yield of hybrids evaluated across four locations during the dry season in 2019.

Hybrid	GY (t/ha)			AD (days)			PH (cm)			EH (cm)			EPP (#)		
	DC	RC	Diff	DC	RC	Diff	DC	RC	Diff	DC	RC	Diff	DC	RC	Diff
**VH1846**	10.46	11.27	0.80	91.65	91.17	-0.48	213.96	212.35	-1.61	102.43	100.67	-1.76	1.04	1.06	-0.02
**VH1899**	10.68	11.08	0.40	91.22	90.98	-0.24	226.38	237.97	11.59	114.66	121.46	6.80	1.07	1.04	0.03
**VH1849**	9.85	9.70	-0.15	90.81	90.91	0.10	235.91	241.04	5.13	114.00	115.48	1.49	1.07	1.04	0.03
**VH1854**	10.16	9.78	-0.38	90.73	91.42	0.70	227.58	233.82	6.23	112.82	115.94	3.13	1.10	1.03	0.08
**VH13729**	9.70	9.60	-0.10	90.60	91.14	0.54	208.92	205.92	-3.00	99.82	96.49	-3.33	1.10	1.09	0.01
**VH16224**	10.52	10.61	0.09	91.40	91.27	-0.13	215.53	217.97	2.45	102.79	103.86	1.07	1.15	1.14	0.00
**VH1657**	7.47	8.40	0.93	90.12	90.53	0.41	205.82	203.82	-2.00	91.40	96.18	4.77	1.07	0.89	0.18
**VH1652**	9.64	10.55	0.91	90.56	91.19	0.63	197.51	198.44	0.93	96.75	105.36	8.61	1.10	1.14	-0.04
**VH151703**	10.74	9.75	-0.99	91.92	91.71	-0.21	204.46	204.05	-0.41	92.01	89.88	-2.14	1.09	1.10	-0.01
**VH122937**	8.44	10.95	2.51	88.32	89.79	1.47	197.47	208.19	10.72	90.23	102.67	12.44	1.06	1.12	-0.06
**VH153492**	9.96	10.16	0.21	90.52	91.03	0.50	209.69	207.96	-1.73	95.86	97.07	1.22	1.11	1.12	-0.01
**VH171219**	8.67	9.53	0.86	90.47	90.76	0.29	217.88	219.59	1.71	97.00	97.64	0.64	1.02	1.09	-0.07
**VH18571**	8.65	7.29	-1.36	91.41	91.69	0.27	219.24	236.90	17.66	101.48	108.24	6.76	1.03	0.76	0.27
**VH16921**	9.04	7.23	-1.81	90.72	90.31	-0.40	224.48	215.44	-9.04	100.61	97.23	-3.38	1.02	0.90	0.11
**VH16923**	10.14	9.92	-0.22	90.05	90.44	0.38	216.01	213.43	-2.58	101.08	94.78	-6.30	1.10	1.05	0.05
**Mean**	9.61	9.72	0.11	90.70	90.96	0.26	214.7	217.1	2.40	100.86	102.86	2.00	1.07	1.04	0.037
**Min**	7.47	7.23		88.32	89.79		197.5	198.4		90.23	89.88		1.02	0.76	
**Max**	10.74	11.27		91.92	91.71		235.9	241.0		114.66	121.46		1.15	1.14	
***p*(T<=t) two-tail**	0.69			0.07			0.17			0.15			0.15		

*p* > 0.05 is non-significant.

DC, direct cross; RC, reciprocal cross; diff, difference.

#, number.

Although maize breeders have long believed that swapping parental roles of maize hybrids does not affect F_1_ performance, published reports directly comparing hybrids and their reciprocals (head-to-head comparison) were not found. Our finding, using a direct comparison of crosses and their reciprocals (head-to-head analysis), is especially noteworthy in light of the previous publications reported above (John et al., 2024; Fan et al., 2014) which highlighted the presence and role of maternal effects in maize based on either a diallel or a North Carolina Design II (line × tester) mating design. The analyses and conclusions based on mating designs are dependent on the group of inbred lines in the study and are designed to parse the mechanism of phenotypic variation into general or specific combining abilities and further into genotypic components which include the maternal, paternal, and reciprocal effects, if appropriately designed. A head-to-head analysis is a direct comparison of the phenotypic performance of the entries in question.

Across the major continents that the CIMMYT Global Maize Program (GMP) is working in, all the hybrid products from Asia and a few from Latin America are single crosses while all hybrids from eastern and southern Africa are three-way crosses. Hence the issue of swapping the parental roles of promising hybrids is relevant in Asia and Latin America for producing commercial F_1_, while in Africa, it is relevant for producing female single crosses. While the CIMMYT internal strategies for hybrid path-to-market are evolving and constantly improving to strengthen SPR (which will help in minimizing the instances of swapping), the current study highlights an important dimension of SPR that is very relevant to CIMMYT, its partners, and the maize hybrid seed industry in general.

The search for heterosis at CIMMYT has been streamlined to that between heterotic groups (HG) A and B which are generally aligned to Tuxpeño and ETO Blanco, respectively (Vasal et al., 1999; Edmeades et al., 2017). Once a promising A x B combination is identified through a systematic stage-gate advancement process, the confirmation of the roles (seed vs. pollen parent) of the inbred lines involved, is dependent on data from the ensuing seed producibility evaluation where seed production data (in small plots) is collected as a preliminary step. This data primarily suffices in giving a broad indication of the roles of the parents *vis-à-vis* synchronization and seed producibility (yield). Although this has helped seed partners in making an informed choice before requesting hybrids for licensing, subsequent commercial scale-up by seed partners necessarily entails evaluations in big plots and seed production isolation pilots. In comparison, evolved breeding programs such as those in the USA, have further refined the Stiff Stalk (SS) vs Non-Stiff Stalk (NSS - e.g., Lancaster) HGs to an extent where, for most hybrids, SS lines are by default the preferred seed parent while the opposing HG (NSS) is the pollen parent; an established system that minimizes the instances where swapping may be needed. CIMMYT’s maize program in Asia and the tropical hybrid maize breeding (especially public) programs in general, can move towards this sophistication and refinement of HGs when germplasm is better organized and more SPR systems are implemented and published.

In conclusion, swapping of female and male parents can be done, at least for the tested single cross hybrids. The unique selling point (USP) of the CIMMYT hybrids results from the product design wherein hybrids perform at least on par with commercial checks under optimal conditions while out-performing them under climatic stresses (drought, heat, and waterlogging). While the inbred parents may confer various levels of stress tolerance to hybrids (based on their combining abilities), the seed production plot itself is always and necessarily under optimal and recommended agronomic management. Swapping parental inbred roles post-identification of superior hybrids (especially with such rare trait combinations) provides a possibility for managing seed producibility issues, if there are any. However, good and detailed SPR is imperative to ensure the success of seed production, especially at large scale, as it requires meticulous planning to ensure that the timing of planting, the management of fields, and the logistics of harvesting and processing align with the specific requirement/s of the hybrid being produced (MacRobert et al., 2014). Swapping between female and male parents should be the last resort and not the norm. Inbred observation nurseries (single row, single replication open pollinated trials) should be adopted for evaluating fixed lines entering Stage 2 (second round evaluation of hybrids). Seed production research should expand to evaluating inbred producibility in bigger isolation plots. Conducting hybrid seed production mini-pilot/s as part of hybrid identification/breeding research before hybrids are commercialized would potentially enhance the speed of product dissemination.

## Data Availability

The data supporting the conclusions of this article will be made available on request through Dataverse by authors, without undue reservation.

## References

[B1] Alvarado G. López M. Vargas M. Pacheco Á. Rodríguez F. Burgueño J.. (2015). META-R (Multi Environment Trail Analysis with R for Windows) Version 6.04 (Mexico: International Maize and Wheat Improvement Center (CIMMYT).

[B2] Colasanti J. Muszynski M. (2009). “The maize floral transition,” in Handbook of Maize: Its Biology. Eds. S. C. Bennetzen Jeff L. Hake (Springer New York, New York, NY), 41–55. doi: 10.1007/978-0-387-79418-1_3

[B3] Dermail A. Suriharn K. Lertrat K. Chankaew S. Sanitchon K. (2018). Reciprocal cross effects on agronomic traits and heterosis in sweet and waxy corn. J. Breed. Genet. 50, 440–460.

[B4] Edmeades G. O. Trevisan W. Prasanna B. M. Campos H. (2017). “Tropical maize (Zea mays L.),” in Genetic Improvement of Tropical Crops. Eds. Campos H. Caligari P. D. S. (Cham: Springer International Publishing), 57–109. doi: 10.1007/978-3-319-59819-2_3

[B5] Fan X. Bi Y. Zhang Y. Jeffers D. Yin X. Kang M. (2018). Improving breeding efficiency of a hybrid maize breeding program using a three heterotic-group classification. Agron. J. 110, 1209–1216. doi: 10.2134/agronj2017.05.0290

[B6] Fan X. M. Zhang Y. D. Yao W. H. Bi Y. Q. Liu L. Chen H. M.. (2014). Reciprocal diallel crosses impact combining ability, variance estimation, and heterotic group classification. Crop Sci. 54, 89–97. doi: 10.2135/cropsci2013.06.0393

[B7] Fleming A. A. Kozelnicky G. M. Browne E. B. (1960). Cytoplasmic effects on agronomic characters in a double-cross maize hybrid. Agron. J. 52, 112–115. doi: 10.2134/agronj1960.00021962005200020017x

[B8] John B. A. Kachapur R. M. Naidu G. Talekar S. C. Rashid Z. Vivek B. S.. (2024). Maternal effects, reciprocal differences and combining ability study for yield and its component traits in maize (Zea mays L.) through modified diallel analysis. Peer J. 12:e17600. doi: 10.7717/peerj.17600 38948201 PMC11212646

[B9] Jumbo M. B. Carena M. J. (2008). Combining ability, maternal, and reciprocal effects of elite early-maturing maize population hybrids. Euphytica 162, 325–333. doi: 10.1007/s10681-007-9618-9

[B10] Kovačević A. Pavlov J. Stevanović M. Grčić N. Mladenović M. Delić N.. (2022). Effects of reciprocal crosses on grain yield and other agronomic traits in maize. Genetika 54, 1365–1374. doi: 10.2298/GENSR2203365K

[B11] MacRobert J. F. Setimela P. S. Gethi J. Regasa M. W. (2014). Maize Hybrid Seed Production Manual. (Mexico, D.F: CIMMYT).

[B12] Mondo V. H. V. Cicero S. M. Dourado-Neto D. Pupim T. L. Dias M. A. N. (2013). Effect of seed vigor on intraspecific competition and grain yield in maize. Agron. J. 105, 222–228. doi: 10.2134/agronj2012.0261

[B13] Moterle L. M. Braccini A. L. Scapim C. A. Pinto R. J. B. Gonçalves L. S. A. do Amaral Júnior A. T.. (2011). Combining ability of tropical maize lines for seed quality and agronomic traits. Genet. Mol. Res. 10, 2268–2278. doi: 10.4238/vol10-3gmr1129 21968766

[B14] Pollmer W. G. Klein D. Dhillon B. S. (1979). Differences in reciprocal crosses of maize inbred lines diverse for protein content. Euphytica 28, 328. doi: 10.1007/BF00056590

[B15] Rezende W. S. Beyene Y. Mugo S. Ndou E. Gowda M. Sserumaga J. P.. (2020). Performance and yield stability of maize hybrids in stress-prone environments in eastern Africa. Crop J. 8, 107–118. doi: 10.1016/j.cj.2019.08.001

[B16] Santos J. F. Dirk L. M. A. Downie A. B. Sanches M. F. G. Vieira R. D. (2017). Reciprocal effect of parental lines on the physiological potential and seed composition of corn hybrid seeds. Seed Sci. Res. 27, 206–216. doi: 10.1017/S0960258517000095

[B17] Vasal S. K. Cordova H. Pandey S. Srinivasan G. (1999). “Tropical maize and heterosis,” in Genetics and Exploitation of Heterosis in Crops. Eds. Coors J. G. Pandey S. (UAS: John Wiley & Sons, Inc), 363–373. doi: 10.2134/1999.geneticsandexploitation

[B18] Zhu M. Tong L. Xu M. Zhong T. (2021). Genetic dissection of maize disease resistance and its applications in molecular breeding. Mol. Breed. 41, 32. doi: 10.1007/s11032-021-01219-y 37309327 PMC10236108

